# From early life to senescence: individual heterogeneity in a long‐lived seabird

**DOI:** 10.1002/ecm.1275

**Published:** 2017-10-26

**Authors:** Rémi Fay, Christophe Barbraud, Karine Delord, Henri Weimerskirch

**Affiliations:** ^1^ Centre d'Etudes Biologiques de Chizé UMR 7372 CNRS/Univ La Rochelle 79360 Villiers‐en‐Bois France

**Keywords:** capture–mark–recapture, delayed density effect, *Diomedea exulans*, finite mixture model, individual quality, population dynamics, Wandering Albatross

## Abstract

Although population studies have long assumed that all individuals of a given sex and age are identical, ignoring among‐individual differences may strongly bias our perception of eco‐evolutionary processes. Individual heterogeneity, often referred to as individual quality, has received increasing research attention in the last decades. However, there are still substantial gaps in our current knowledge. For example, there is little information on how individual heterogeneity influences various life‐history traits simultaneously, and studies describing individual heterogeneity in wild populations are generally not able to jointly identify possible sources of this variation. Here, based on a mark–recapture data set of 9,685 known‐aged Wandering Albatrosses (*Diomedea exulans*), we investigated the existence of individual quality over the entire life cycle of this species, from early life to senescence. Using finite mixture models, we investigated the expression of individual heterogeneity in various demographic traits, and examined the origin of these among‐individual differences by considering the natal environmental conditions. We found that some individuals consistently outperformed others during most of their life. In old age, however, the senescence rate was stronger in males that showed high demographic performance at younger ages. Variation in individual quality seemed strongly affected by extrinsic factors experienced during the ontogenetic period. We found that individuals born in years with high population density tended to have lower performances during their lifespan, suggesting delayed density dependence effects through individual quality. Our study showed that among‐individual differences could be important in structuring individual life history trajectories, with substantial consequences at higher ecological levels such as population dynamics.

## Introduction

Population studies have long implicitly assumed that all individuals of a given sex and age are identical. In reality, among‐individual differences are ubiquitously observed within populations that consist of phenotypically diverse individuals (Bolnick et al. 2011). Neglecting this individual heterogeneity may affect our understanding of empirical observations, leading to spurious conclusions. For instance, processes acting at the individual scale may be very different from the average patterns observed at the population scale (Vaupel and Yashin 1985). If underperforming individuals are more likely to die young, the age‐specific performance estimated at the population level could actually increase with age, even though the opposite pattern can be operative at the individual scale. Ignoring among‐individual differences can therefore hide fundamental biological processes such as senescence (Nussey et al. 2008) or trade‐offs among life‐history traits (Hamel et al. 2009a). The consequences of individual heterogeneity are not limited to within‐individual patterns, as it also affects population dynamics (Benton et al. 2006). This means that two populations with identical initial average vital rates but different levels of individual heterogeneity will show different dynamics, leading to divergent asymptotic population growth rates and population stability (Kendall et al. 2011). Thus, the working assumption often made in population studies that all individuals are identical within a population, or a component of a population, is a strong assumption that could bias our perception of ecological processes.

Although individual differences leading to contrasting life‐history trajectories constitute a fundamental condition of the theory of natural selection (Darwin 1859), heterogeneity among individuals appears to be a difficult concept to define and a hard trait to measure (Wilson and Nussey 2010, Bergeron et al. 2011, Cam et al. 2012). This elusive aspect of individual heterogeneity has been explicitly formulated through the expression of latent or unobservable individual heterogeneity (Cam et al. 2002). Depending on their field and the life‐history traits studied, authors have tackled individual heterogeneity in different ways using various terms such as frailty (Vaupel et al. 1979), state (McNamara and Houston 1996), or quality (Wilson and Nussey 2010), but all these authors agree that intrinsic individual factors, independently of age and sex, consistently modify reproduction and survival performances. Recently, this biological interpretation of individual heterogeneity was questioned in a series of articles, adding a new level of complexity (Tuljapurkar et al. 2009, Steiner and Tuljapurkar 2012). These authors stated that chance alone accounted for most of the observed variation in individual life histories in the wild, without the necessity of evoking underlying individual heterogeneity in vital rates. They used the term dynamic heterogeneity to describe variation in individual performances that arise from the stochastic nature of individual life trajectories, equivalent to a Markovian transition among biological states. However, recent research has called attention to weaknesses in the inference methods used in these studies, which raises some doubts about their conclusions (Plard et al. 2012, Bonnet and Postma 2016, Cam et al. 2016, Authier et al. 2017). Both fixed, i.e., quality, and dynamic heterogeneity are probably important to explain the high variability in individual life history trajectories (Cam et al. 2012, Plard et al. 2012, Chambert et al. 2013, Jenouvrier et al. 2017) and more importantly, both sources of variability need to be considered simultaneously to ensure reliable inferences (Cam et al. 2016).

The observation of fixed individual heterogeneity can be traced back to the seminal work of Lack (1954), who noted consistent differences in clutch size among individuals that shared the same environment. In the last decades, numerous studies based on long‐term individual monitoring of wild populations documented unexpected correlation among vital rates, showing that some individuals consistently outperformed others. Contrary to the life‐history theory expectation of trade‐offs among life‐history traits (Stearns 1992), positive correlation was found between breeding success and survival (Bérubé et al. 1999, Cam et al. 2002), previous reproductive state and current breeding probability (Hamel et al. 2009a, Jenouvrier et al. 2015), and secondary sexual character allocation and survival (Bergeron et al. 2008), while the age at first and last reproduction could be negatively related (Charmantier et al. 2006, Aubry et al. 2011). All these results are difficult to explain without invoking individual differences. Theoretically, individuals showing consistently high demographic performance are expected to acquire more resources (Van Noordwijk and de Jong 1986). This prediction was empirically supported by studies demonstrating that consistent among‐individual heterogeneity in reproductive and survival performance was related to diet specialization and foraging performance (Annett and Pierotti 1999, Lescroël et al. 2010). Thus, at least part of the variation in individual life‐history trajectories may occur because of individual differences affecting energy acquisition (Reznick et al. 2000).

On the other hand, it could be argued that spatial heterogeneity alone may create such individual variation in life trajectories, without evoking intrinsic differences among individuals. Indeed, if individuals in a population experience different environmental conditions, this can lead to individual heterogeneity in demographic rates (Griffen and Norelli 2015). However, there is strong evidence that the local individual distribution of mobile organisms in heterogeneous habitats is not random at all. Spatial distribution seems closely related to individual features (Coulson 1968, Camacho et al. 2013); more competitive individuals may have access to high quality habitats, constraining subordinate individuals to settle in marginal habitats (van de Pol et al. 2007, Oro 2008). Therefore, spatial heterogeneity makes the comprehension of among‐individual variation even more difficult, but does not bring into question the concept of fixed heterogeneity as intrinsic individual differences. Consistently, individual heterogeneity has been documented in laboratory populations under controlled environments and in wild populations in non‐territorial species such as pelagic seabirds (Cam et al. 2012, Fay et al. 2016).

Although individual heterogeneity has received increasing research attention in the last decades, there are still substantial gaps in current knowledge. For example, it is generally accepted that among‐individual differences arise from both genetic and non‐genetic factors, but studies describing individual heterogeneity in wild populations have not attempted, in the vast majority of cases, to identify possible sources of this variation. Furthermore, owing to the difficulties associated with measuring individual heterogeneity, in particular with the problem of identifying parameters associated with multiple random effects (Knape et al. 2011), most of the studies investigated heterogeneity in one or two specific traits. Thus, there is little information on individual heterogeneity for various life‐history traits taken into consideration simultaneously (Moyes et al. 2009).

Given that individual heterogeneity has been addressed in various ways leading to confusion and ambiguity (Wilson and Nussey 2010), we need to accurately define our view of among‐individuals heterogeneity. The comprehensive approach adopted in this study is often referred to as individual quality. We retain the working definition of Wilson and Nussey (2010), “an axis of among‐individual heterogeneity that is positively correlated with fitness.” Here, we consider that individual quality is an unmeasured feature of phenotypes and thus a fixed property of each individual (Cam et al. 2012). The static character of individual quality was supported through individuals maintaining higher performance levels across a large range of environmental conditions. Under extreme conditions, it appears that individual quality is not reversed or attenuated but exacerbated (Chambert et al. 2013, Jenouvrier et al. 2015).

Long‐lived seabirds are a convenient model for population demographic studies because they breed in large colonies and show high philopatry. They are also suitable model species for studies on individual heterogeneity owing to their long lifespan that allow us to investigate the repeatability of demographic performance over time. Furthermore, their non‐territorial foraging behavior relaxes the potential effect of spatial heterogeneity. Here, based on a mark–recapture data set of 9,685 known‐aged Wandering Albatrosses (*Diomedea exulans*), we investigate the existence of individual quality over the whole life cycle, from fledging to senescence. We use finite mixture models to deal with the elusive character of individual quality while taking both dynamic and fixed heterogeneity into account. Our main objective was to gain insight into the expression of individual heterogeneity among various demographic traits, including early‐life survival, recruitment rate, breeding success at first reproduction, adult breeding probability, adult breeding success, and adult survival. Since consistent individual variation in vital rates could come from early‐life environment having long‐term effects on phenotypes (Lindström 1999), adverse conditions experienced during ontogeny may affect future mortality and fecundity. Thus, by estimating cohort average quality, we investigated the origin of this heterogeneity through natal environmental conditions considering climatic factors and population size. We predicted (1) that inferences made from mixture models would support the quality hypothesis with some individuals consistently outperforming others, (2) that there is cohort variation in average quality, i.e., cohort specific probability to be associated to quality groups of the mixture model, and (3) that a part of these variations in average quality could be explained by the early‐life environmental conditions.

## Materials and Methods

### Study species and field methods

We studied the Wandering Albatross population of Possession Island in the Crozet Archipelago (46° S; 52° E), southern Indian Ocean, from 1965 to 2013. Monitoring started in 1960, but all chicks were ringed each year with a stainless steel band just before fledging from 1965. Each year, from early to mid‐December, pre‐breeding adults were checked for bands over the whole island. From mid‐January (just after egg laying is resumed) to mid‐February, at least three visits were made every 10 days to identity the two members of each pair and to ascertain their breeding status. All unmarked individuals were ringed with a uniquely numbered stainless steel‐band. In mid‐April, June, and August, nests were checked and the chick status (alive/dead) recorded. During all visits, non‐breeding individuals (mainly immatures) were searched for and their identities determined (from band numbers) when possible. From mid‐September to mid‐October fledglings were ringed. Sex assignments were based on field observations (i.e., sexual size and plumage dimorphism, mating behaviors) and since 1999 on genetic analyses (Appendix S1).

Wandering Albatrosses show a typical slow life history strategy with high adult survival rates and low productivity (i.e., quasi biennial reproduction and clutch size limited to one egg without replacement laying). Fledglings leave the colony alone, and remain at sea continuously until they return to their colony of birth from 3 yr old at the earliest (Weimerskirch 1992). Recruitment occurs from 6 to 15 yr old. Females recruit earlier than males, but both sexes show increasing recruitment probability until 9–10 yr old followed by a decrease at older age (Fay et al. 2015a).

### General model

The capture–recapture data set was modeled with the same multi‐event model (Pradel 2005) structure used by Fay et al. (2016) on the same population. Briefly, the model comprised seven states (one immature state, five adult states, and the state dead; Appendix S3: Fig. S1) and five events. The following four states were observable: the pre‐recruitment state (PrR) to consider individuals during the period of immaturity, the successful breeder state (SB) for breeders fledging chicks, the failed breeder state (FB) for breeders whose chicks did not survive until fledging, and the recruited non‐breeder state (NB) for individuals that have recruited into the population (i.e., bred at least once) and were present as non‐breeders at the colony. Two unobservable states, the post successful breeder state (PSB) and post failed breeder state (PFB), were used to model the sabbatical years spent at sea after reproduction. Biological constraints were applied regarding sex, age, and time dependence, based on previous results from Fay et al. (2015a), as follows. The PrR state was divided into two stages: the juvenile stage, which was an unobservable stage, corresponding to the first 2 yr of life spent continuously at sea (i.e., 1‐ or 2‐yr‐old individuals were never observed at the colony), and the immature stage corresponding to non‐recruited birds older than 2 yr that started to visit the colony and could be potentially observed. The immature stage was decomposed into three age classes: 3–8 yr, 8–13 yr, and >13 yr corresponding to the most parsimonious age structure to model the progressive change of survival rate in early life. Juvenile survival was set to be cohort dependent and both juvenile and immature survival rates were assumed to be sex dependent. From the age of 6, immature birds can recruit into the breeding population, i.e., lay an egg for the first time. Recruitment rate depended both on sex and age and was constrained to be constant after age 10. For adult states, survival was assumed to be sex and state dependent distinguishing between NB and the others states (Pardo et al. 2014). Actuarial senescence was estimated by modeling adult survival as a quadratic function of age starting at the average age of primiparity (10 yr; Pardo et al. 2013). Due to excessive computation time and convergence difficulties, we did not investigate more complex senescence patterns. Actuarial senescence rate varied with sex (Pardo et al. 2013) but was constrained to be equal among states to avoid estimation difficulties due to data sparseness at older ages. Transitions were set to be state dependent, but since Wandering Albatrosses are monogamous and both sexes exhibit quasi‐biennial breeding, transitions were constrained to be equal between the sexes. Fay et al. (2016) provide further details on the general starting model and parametrization.

### Heterogeneity

**Figure 1:**
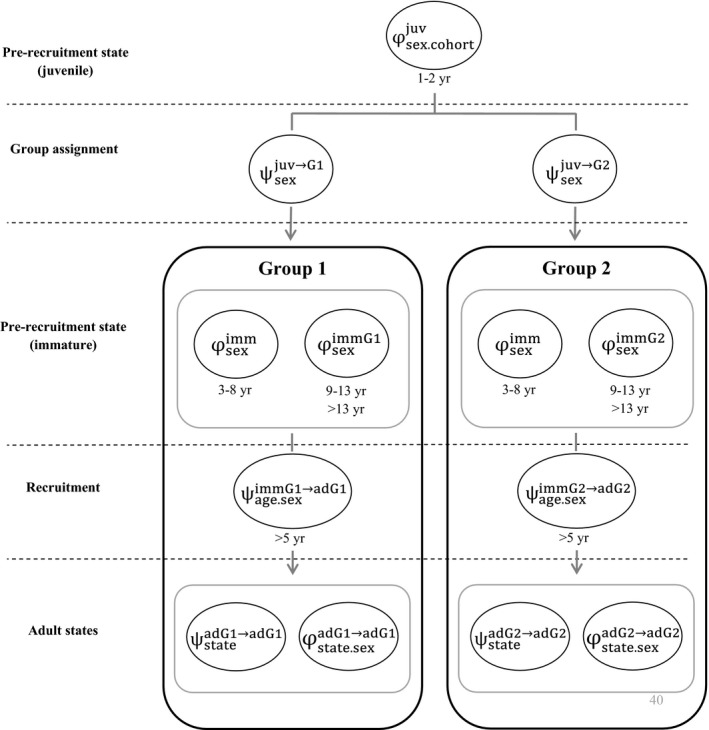
Graph summarizing the finite mixture model used. After juvenile mortality took place ($\varphi_{\mathrm{sex}.\mathrm{cohort}}^{\mathrm{juv}}$), individuals may transition into group 1 or 2 ($\psi_{\mathrm{sex}}^{\mathrm{juv}\to Gj}$, *j* = 1, 2) according to their life history trajectory, including immature survival ($\varphi_{\mathrm{sex}}^{\mathrm{immG}j}$), recruitment probability ($\psi_{\mathrm{age}.\mathrm{sex}}^{\mathrm{immG}j\to \mathrm{adG}j}$), adult breeding parameters ($\psi_{\mathrm{state}}^{\mathrm{adG}j\to \mathrm{adG}j}$), and adult survival ($\varphi_{\mathrm{state}.\mathrm{sex}}^{\mathrm{adG}1\to \mathrm{adG}1}$).

We used finite mixture models to investigate individual heterogeneity (Pledger et al. 2003). These models, which use clustering among individuals, allow latent heterogeneity to be estimated in the absence of a priori criteria such as body size or other morphological traits (Hamel et al. 2016). Based on the general model, we investigated individual heterogeneity in survival and transition parameters for both immature and adult states. Each immature and mature state (i.e., PrR, SB, FB, NB, PSB, PFB) was duplicated constituting two groups with specific survival and transition probabilities (Fig. 1). The number of groups is a critical issue for mixture models but previous work by Pledger suggested that two groups were sufficient for hidden variables following a unimodal distribution (Pledger 2005). In our case, assuming a unimodal distribution for individual quality, as often suggested for frailty, seemed reasonable since we defined it as an individual phenotypic characteristic that is determined by many factors acting simultaneously.

We allowed vital rate parameters to vary between the two components of the mixture model. Hence, according to the individual quality theory, we expect that the demographic rates of the two groups will differ systematically such that one group will have consistently higher vital rates than the other. Individuals are not pre‐assigned a priori to a particular group, but instead are assigned probabilistically to each group based on their life history trajectory, i.e., how they survived and transitioned among states. Finite mixture models are expected to reduce unexplained residual variance by gathering within each group individuals sharing the most similar vital rates. In accordance with the conceptual view of individual quality adopted in this study, individual quality assignment is positively correlated, but not confounded, with fitness. Indeed, a higher average vital rate at the group scale does not systematically imply higher lifetime reproductive success at the individual scale. Our model took into account both fixed and dynamic heterogeneity as individual life trajectories were modeled as a Markovian process (dynamic component) within each mixture (fixed component). Hence, dynamic heterogeneity may lead lifetime reproductive success to vary importantly around the average performance within each group. This variability is consistent with our theoretical concept of quality as a fixed property of a phenotype that exists a priori, regardless of the individual's realized history.

Preliminary analyses indicated that juvenile survival may drive individual assignment toward group 1 or 2 owing to the high variability of this parameter (Fay et al. 2015a). Thus, group assignment was performed after modeling cohort‐specific juvenile survival (Fig. 1). At this point, each individual has a probability $\psi_{\mathrm{sex}}^{\mathrm{juv}\to G1}$ to transition into group 1 and a probability $\psi_{\mathrm{sex}}^{\mathrm{juv}\to G2}\left(=1-\psi_{\mathrm{sex}}^{\mathrm{juv}\to G1}\right)$ to transition into group 2, as a function of sex. Once assigned to group G*j* (=1, 2), each immature individual has survival probabilities $\phi_{\mathrm{age}.\mathrm{sex}}^{\mathrm{immG}j}$ and recruitment probabilities $\psi_{\mathrm{age}.\mathrm{sex}}^{\mathrm{immG}j\to \mathrm{adG}j}$. For parameters to be identifiable, age 3–8 yr immature survival was constrained to be equal between groups. After recruitment, each individual has transition probabilities $\psi_{\mathrm{state}}^{\mathrm{adG}j\to \mathrm{adG}j}$and survival probabilities $\phi_{\mathrm{sex}.\mathrm{state}}^{\mathrm{adG}j}$ (Fig. 1). Once an individual is assigned to group *j*, defined by the set of parameters presented above, it cannot transition to the other group. Finally, to gain insight about the origin of individual heterogeneity, we estimated cohort‐specific assignment to each mixture, i.e., $\psi_{\mathrm{sex}+\mathrm{cohort}}^{\mathrm{juv}\to Gj}$. Environmental conditions experienced early in life may have long‐term effects on individual fitness (Lindström 1999). Thus, we investigated the effect of natal environment conditions on the cohort‐specific probability of belonging to group *j*. We selected two variables that are expected to affect ontogeny at the pre‐fledging and/or post‐fledging stage: the sea surface temperature on paternal foraging grounds during chick rearing, and the population density in the year of birth estimated by the size of the annual breeding population (Fay et al. 2015a). We fitted the logistic model $\text{logit}\left(\psi_{\mathrm{sex}+\mathrm{cohort}}^{\mathrm{juv}\to Gj}\right)=\beta_{0}+\beta_{1}\times x_{n}$_,_ where $\psi_{\mathrm{sex}+\mathrm{cohort}}^{\mathrm{juv}\to Gj}$ is the cohort‐specific transition probability into group *j*, β_0_ is an intercept parameter, β_1_ is a slope parameter, and *x*_*n*_ is the covariate *x* the year of birth for the cohort *n*. We tested both linear and quadratic effects for group assignment. Significance of relationships was assessed by an analysis of deviance test with a Fisher‐Snedecor distribution (ANODEV; Grosbois et al. 2008). The percentage of variation that was explained by a covariate (*r* ^2^) was estimated as $r^{2}=[\left(\text{Dev}\left(F_{\mathrm{cst}}\right)-\text{Dev}\left(F_{\mathrm{cov}}\right)\right]/\left[\text{Dev}\left(F_{\mathrm{cst}}\right)-\text{Dev}\left(F_{t}\right)\right]$ (Skalski 1996).

To summarize, our initial finite mixture model was

$$\begin{array}{cc} & \Phi_{{\text{age}}_{\left(1\;\text{to}\;2\right)}.\text{cohort}+\text{sex}}^{\text{juv}}\Psi_{\text{sex}}^{\text{juv}\to G1}\Phi_{{\text{age}}_{\left(3\;\text{to}\;8\right)}.\text{sex}}^{\text{imm}}\Phi_{{\text{age}}_{\left(9\;\text{to}\;13\right)},{\text{age}}_{\left(>13\right)}.\text{sex}}^{\text{immG}1}\\ & \Phi_{{\text{age}}_{\left(9\;\text{to}\;13\right)},{\text{age}}_{\left(>13\right)}.\text{sex}}^{\text{immG}2} \end{array}$$

$\Psi_{\mathrm{age}.\mathrm{sex}}^{\mathrm{immG}1\to \mathrm{adG}1}\Psi_{\mathrm{age}.\mathrm{sex}}^{\mathrm{immG}2\to \mathrm{adG}2}$ for the immature component and $\Phi_{\mathrm{age}+\mathrm{sex}.\mathrm{state}}^{\mathrm{adG}1}\Phi_{\mathrm{age}+\mathrm{sex}.\mathrm{state}}^{\mathrm{adG}2}\Psi_{\mathrm{state}}^{G1\to G1}\Psi_{\mathrm{state}}^{G2\to G2}p_{\mathrm{age}}^{\mathrm{pre}}p_{\mathrm{state}}^{\mathrm{ad}}$ for the adult component, where the juvenile (juv) survival probability (Φ) was sex and cohort dependent, the immature (imm) survival probability was age and sex dependent, the adult (ad) survival probability was age, sex, and state dependent, the probability of transitioning (Ψ) from immature to adult through recruitment was age and sex dependent, the probability of transition into the group 1 (G1) given recruitment was sex dependent, the pre‐recruitment capture probability (*p*) was age dependent, and the adult capture probability was state dependent. In this model notation, the symbol “.” indicates interactive effects, “+” indicates additive effects, “1 to 2”, “3 to 8” and “9 to 13” indicate that age classes were grouped and “>13” indicates that age classes were grouped after 13 yr.

### Breeding performance and reproductive value

Because our model had full state‐dependent transitions, we did not have direct access to breeding probability and breeding success estimates. Both were calculated from the outputs of the multievent modeling. For a given state, breeding probability, i.e., the probability to have an egg, is the probability to be in state SB (successful breeder) plus the probability to be in state FB (failed breeder). The breeding success is the ratio between the probability to be in state SB and the breeding probability (whether successful or not). 95% confidence intervals for these derived estimates were obtained with a bootstrap method (Appendix S2). We did not estimate the breeding performance of previous Successful Breeder (SB) since most of the individuals in this state take a sabbatical year at the next occasion, i.e., they transition toward the PSB state, nor previous post‐failed breeder (PFB) because too few individuals transit in this state.

Finally, sex‐specific reproductive values were estimated for each group to summarize the overall lifetime performance. Here reproductive value is the number of offspring an individual can expect to obtain during the current year and the remainder of its life. Using average age‐ and sex‐specific survival and reproductive performance estimates (the average number of chicks produced by year), we calculated the reproductive value with the following equation:

$${\text{RV}}_{a}=\sum_{x=a}^{\mathrm{ALR}}\frac{l_{x}}{l_{a}}m_{x}$$

where RV_*a*_ is the reproductive value at the age *a*, ALR is age at last reproduction, *l*_*x*_ and *l*_*a*_ are the probabilities to survive until the age respectively of *x* and *a*, and *m*_*x*_ is the average number of chicks produced by an individual of age *x* (Stearns 1992).

### Model selection and goodness of fit

Model selection was done using Akaike Information Criteria (AIC, Burnham and Anderson 2002) to test sex and group specific actuarial senescence patterns and sex and cohort effects on group assignment $\psi_{\mathrm{sex}+\mathrm{cohort}}^{\mathrm{juv}\to G2}$. Models with ΔAIC < 2 were not considered meaningfully different (Burnham and Anderson 2002). All models were run using the program E‐SURGE (Choquet et al. 2009b).

There is no test available to assess the goodness of fit (GOF) of multi‐event models. We thus performed GOF tests using program U‐CARE (v.2.3.2; Choquet et al. 2009a) on a simplified data set, which distinguished solely successful breeders from failed breeders and randomly assigned a reproductive status, i.e., failed or successful, to each individual for which no information was available (Pradel 2005). Results suggested slight overdispersion with a variance inflation factor ($\hat{c}$) of 1.37. Since a substantial part of the overdispersion not captured by the simple multi‐state model used for GOF testing was likely captured in our finite mixture model controlling for age, cohort, and individual heterogeneity, we assumed that our general model fitted the data correctly.

## Results

A two‐class mixture model strongly improved our general homogenous model (Table 1, M1 vs. M2, ΔAIC = 1026.3) suggesting important individual heterogeneity in this population. On average, 40% ± 3% of birds belonged to the first group and 60% ± 3% to the second group. However, model selection suggested sex‐specific partitioning into groups (Table 1, M2 vs. M3, ΔAIC = 39.8), with 53% ± 4% of females compared to 30% ± 3% of males being assigned to the first group. In accordance with the concept of individual quality, individuals from the first group showed consistently higher demographic performance than individuals from the second group.

**Table 1:** Testing for individual heterogeneity in Wandering Albatrosses at Crozet from 1965 to 2012

No.	Model	*k*	Dev	AIC	ΔAIC
M1	ψ^juv→G1^ (null)	116	95,835.8	96,067.8	1074.5
M2	ψ^juv→G1^ (cst)	167	94,707.5	95,041.5	48.2
M3	ψ^juv→G1^ (sex)	169	94,663.7	95,001.7	8.4
**M4**	ψ^**juv→G1**^ **(sex + coh)**	**202**	**94**,**589.3**	**94**,**993.3**	**0**
M5	ψ^juv→G1^ (sex.coh)	235	94,545.0	95,015.0	21.7

*Notes*

M1 (null) and M2 (constant [cst]) test respectively for absence or presence of individual heterogeneity. M3 (sex) tests for sex‐specific group assignment. M4 and M5 (coh) test for cohort‐specific group assignment with respectively additive (+) and interactive effects (.). ψ^juv→G1^ is the probability to transition from juvenile to stage G1. Results include the number of mathematical parameters (*k*), the deviance (Dev), Akaike information criterion value (AIC), and the difference in AIC value of the model with respect to the best model (ΔAIC). The best supported model is shown in boldface type.

**Figure 2:**
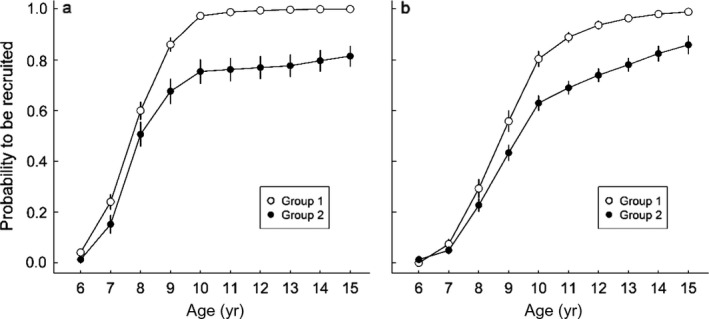
Heterogeneity of cumulated probability to be recruited according to age and sex for (a) females and (b) males of the Wandering Albatross population of Crozet. Values (estimates ± SE) were calculated from age‐specific recruitment and survival probabilities (model 4, Table 1) through bootstrapping methods (1,000 simulations).

**Figure 3:**
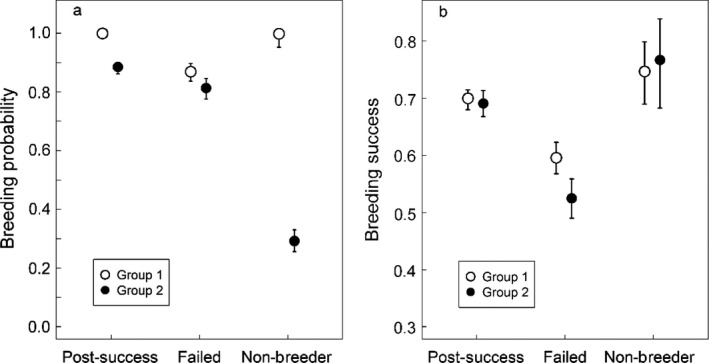
Heterogeneity in (a) breeding probability and (b) breeding success considering the previous breeding state for adult Wandering Albatrosses from Crozet Island from 1965 to 2012. Estimates were calculated from the outputs of M4 (Table 1) and 95% confidence intervals were obtained with a bootstrap method.

**Figure 4:**
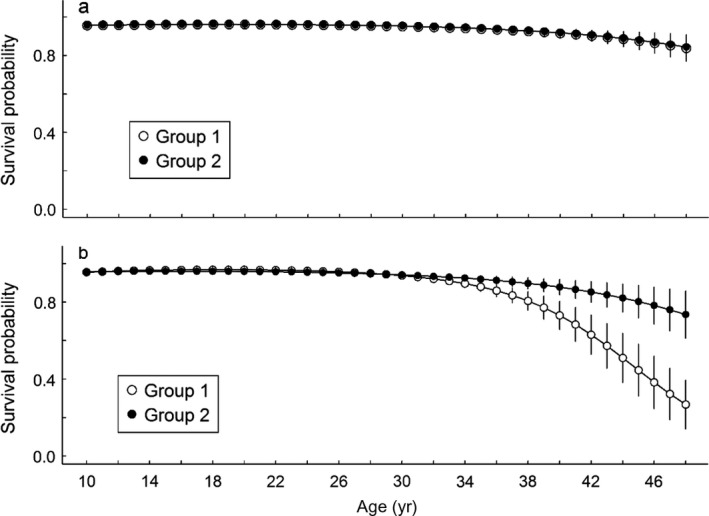
Heterogeneity of age‐specific survival probability for breeders and post‐reproductive breeders (a) females and (b) males for the Wandering Albatross population at Crozet. Estimates (±se) were obtained from model averaging the three best competitive models (M2, M4, and M5, Table 3).

Immature individuals from group 1 showed both higher survival probability (Table 2) and higher recruitment rate (Fig. 2). Independent of age and sex, recruitment probability was consistently higher in group 1 (Appendix S3: Fig. S2), leading to full recruitment after 10 yr for females and at 14 yr for males. In contrast, about 20% of immature individuals from the second group had not recruited at 15 yr of age. Furthermore, given recruitment, we found that breeding success at first reproduction was higher in the first group (0.50 ± 0.03) compared to the second (0.42 ± 0.03). During adulthood, individuals from group 1 were breeding more often regardless of their previous state. Previously failed breeders from group 1 also had higher breeding success than previously failed breeders from group 2 (0.60 ± 0.03 vs. 0.53 ± 0.04, respectively, Fig. 3). We note that previous post‐success and non‐breeder individuals have similar breeding success between groups. Finally, the average annual probability of producing a chick, i.e., both breeding probability and success probability given reproduction, weighted by the proportion of individuals in each state was 0.63 in group 1 and 0.38 in group 2. Contrary to reproductive performances, we found less evidence for heterogeneity in adult survival. Survival probability was equal between groups for both males and females until age 30 yr. After this, age senescence appeared in both sexes, with a progressive decrease of survival probability. Senescence seemed group specific for males only, suggesting heterogeneity for the male (but not female) senescence rate. Although heterogeneity for the male senescence rate was included in the best‐supported model, it was not meaningfully different from a model without heterogeneity (Table 3, M2 vs. M5, ΔAIC = 1.63). However, we note that removing heterogeneity for the female senescence rate reduced AIC by 4 points, whereas removing heterogeneity for male senescence rate increased AIC by 1.2 points. We found support for sex‐specific senescence rates (Table 3, M3 vs. M1, ΔAIC = 3.06), but senescence rate of males from group 2 was indistinguishable from female senescence rate (Table 3, M5 vs. M4, ΔAIC = −0.21) contrary to senescence rate of males from group 1 (Table 3, M6 vs. M4, ΔAIC = 5.41). Model‐averaged estimates (Table 3, M2, M4, and M5) clearly suggested faster decrease of survival for males from group 1. For this group, survival probability fell sharply from 0.96 before 30 yr of age to less than 0.50 after 45 yr of age. Over the same age range, survival for males from group 2 declined gradually to 0.80 (Fig. 4).

**Table 2:** Heterogeneity of immature survival for the Wandering Albatross population from Crozet

Age class	Sex	Estimate		
		Group 1	Group 2	Without heterogeneity (Fay et al. 2015a)
9–13 yr	male and female	0.999 ± 0.001	0.960 ± 0.019	0.980 ± 0.008
>13 yr	male	0.940 ± 0.115	0.869 ± 0.039	0.876 ± 0.036
>13 yr	female		0.922 ± 0.029	0.768 ± 0.061

*Notes*

Estimates (±SE) are from model 4 (Table 1). Survival estimates for >13‐yr‐old immature females from group 1 are not estimable because full recruitment has been reached at this age (Fig. 2).

**Table 3:** Model selection for the effects of sex and heterogeneity on survival senescence rate for Wandering Albatrosses at Crozet (1965–2012)

No.	Model	*k*	Dev	AIC	ΔAIC
M1	Fe.G + Ma.G	172	94,672.96	95,016.96	4.21
M2	Fe + Ma	168	94,678.38	95,014.38	1.63
M3	G	168	94,684.02	95,020.02	7.27
M4	Fe + Ma.G	170	94,672.96	95,012.96	0.21
**M5**	**(Fe = Ma.G2) + Ma.G1**	**168**	**94**,**676.75**	**95**,**012.75**	**0**
M6	(Fe = Ma.G1) + Ma.G2	168	94,682.37	95,018.37	5.62
M7	Fe.G + Ma	170	94,678.18	95,018.18	5.43
M8	Fe.G1 + (Fe.G2 = Ma)	168	94,681.06	95,017.06	4.31
M9	Fe.G2 + (Fe.G1 = Ma)	168	94,682.67	95,018.67	5.92

*Notes*

Fe, female; Ma, male; G, heterogeneity group effect; *X* = *Y* mean that the senescence rate of *X* is constrained to be equal to the senescence rate of *Y*. For example, (Fe = Ma.G2) + Ma.G1 indicates that senescence rate of females from both heterogeneity groups (1 and 2) is constrained to be equal to the senescence rate of males from group 2, but is different from the senescence rate of males from group 1. *K*, number of parameters; Dev, deviance; AIC, Akaike information criterion, and the difference in AIC value of the model with respect to the best model (ΔAIC). The best supported model is shown in boldface type.

However, young males from group 1 still have a higher reproductive value than males from group 2. At 9 yr of age, the reproductive value was 5.90 for group 1 compared to 3.28 for group 2. Similar results were obtained for 9‐yr‐old females, with a reproductive value of 5.70 for group 1 and 3.36 for group 2.

**Figure 5:**
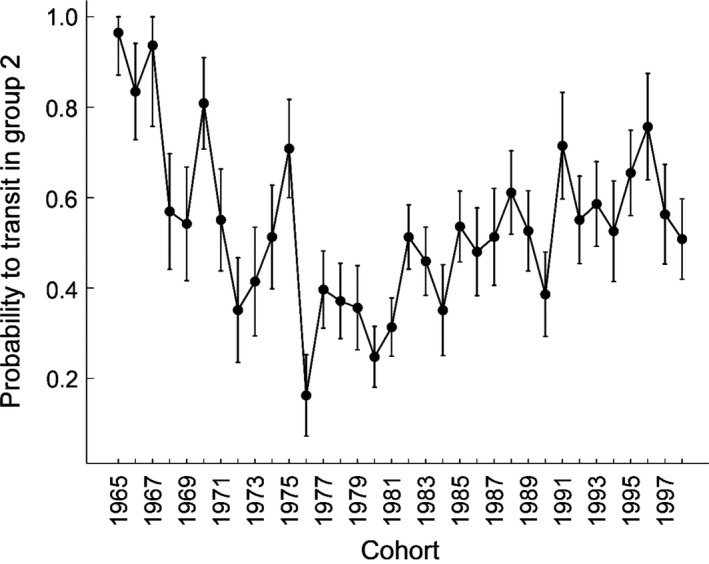
Cohort heterogeneity: cohort‐specific probability to transit in group 2. Estimates (±SE) were derived from model M4 (Table 1).

Finally, our results support additive cohort‐specific probability to belong to one of the two groups (Table 1, M3 vs. M4, ΔAIC = 8.4). The probability to transit to group 2 decreased from about 0.80 in the late 1960s to 0.25 in the early 1980s, and then increased progressively to around 0.60 in the late 1990s (Fig. 5). Investigating the early‐life conditions that could explain this variation, we found a positive effect of population size during the year of birth on the probability to belong to group 2 (Table 4, M7 *F*_cst/co/*t*_ = 3.60, df = 32, *P* = 0.04; Appendix S3: Fig. S3). This variable explained 18% of cohort variations. Individuals born in years of low population density thus tended to have higher demographic performance throughout their life (except for senescent males). We found no evidence of an effect of the early‐life sea surface temperature on the probability to belong to one of the two groups (Table 4).

**Table 4:** Testing the effect of early‐life environmental conditions on the cohort‐specific probability to belong to group 2 (low quality individuals) for the Wandering Albatross population at Crozet

No.	Model	Dev	*F*	df	*P*	*r* ^2^	Slope [95% CI]
**M7**	**ψ** ^**juv→G2**^ **(sex + N)**	**94**,**650.0**	**3.60**	32	**0.04**	**0.18**	**+0.33 [+0.14; +0.52]**
M8	ψ^juv→G2^ (sex + N + N^2^)	94,649.5	0.13	31	0.88		
M9	ψ^juv→G2^ (sex + SSTA)	94,606.0	0.04	32	0.96		
M10	ψ^juv→G2^ (sex + SSTA + SSTA^2^)	94,603.3	0.93	31	0.45		
M11	ψ^juv→G2^ (sex + N + SSTA)	94,650.0	0.01	32	0.99		
M12	ψ^juv→G2^ (sex + N + SSTA + SSTA^2^)	94,648.2	0.14	31	0.93		

*Notes*

Results include the deviance (Dev), the statistic *F*_cst/co/*t*_ testing the null hypothesis that the focal environmental variable has no effect on transition probability to group 2, the percentage of variation explained by the covariates (*r* ^2^), and the 95% CI of the slope for linear relationships. Environmental covariates, i.e., population size as approximated by the breeding population size (*N*) and sea surface temperature anomaly (SSTA), were standardized. Models with statistically significant covariate effects at the level of 5% are shown in boldface type.

## Discussion

This study highlighted strong variation in life‐history trajectories and the importance of individual quality to explain these differences. In agreement with the predictions from the individual quality hypothesis, mixture models showed positive covariation among various demographic traits, indicating that individuals had consistent demographic performance throughout their lives. Furthermore, cohort specific variation in demographic traits suggested that individual quality could be determined by the environment experienced in early life. We found that individuals born in years with high population densities tended to have lower performance during their lifespan, suggesting delayed density dependence effects acting through individual quality.

### Individual heterogeneity and the individual quality hypothesis

As predicted by the individual quality hypothesis, we found positive relationships among different individual fitness components throughout the life cycle. From early life to the adult stage, we found positive relationships between immature survival, recruitment rate, breeding success at recruitment, adult reproductive probability and adult breeding success probability given reproduction. In early life, consistent among‐individual differences were already visible through the positive covariation between immature survival and recruitment, as previously documented in the Pacific Black Brant *Branta bernicla nigricans* (Lindberg et al. 2013). In long‐lived species, survival may decline in the oldest immature age classes (Desprez et al. 2014, Fay et al. 2015a), suggesting that individuals delaying recruitment are individuals with lower intrinsic survival probability, i.e., lower quality (Fay et al. 2016). This result is consistent with the idea that individual quality is determined very early in life through fixed intrinsic characteristics. In our case, it appears unlikely that individual quality will be confused with spatial heterogeneity. As a pelagic species, the Wandering Albatross covers extensive areas at low energetic cost using favorable winds, showing non‐territorial foraging behavior (Weimerskirch et al. 2000). Their ability to forage widely allows them to avoid less favorable local conditions and ensures that, once controlled for the effects of age and sex, all individuals potentially have access to the same habitat.

Young individuals with both higher recruitment rate and higher breeding success at their first reproduction tended to also have higher chick productivity during adulthood. Similarly, higher first‐year survival probability was associated with higher adult performances in the Red‐billed Chough *Pyrrhocorax pyrrhocorax* (Reid et al. 2006), earlier age at first reproduction was associated with higher adult survival and reproductive performances in female reindeer *Rangifer tarandus* (Weladji et al. 2008) and female Mute Swan *Cygnus olor* (McCleery et al. 2008). Thus, as in other long‐lived species, some individuals outperformed others without apparent cost during most of the adult life (Hamel et al. 2009a).

Our results indicate that a higher proportion of females than males were assigned to the higher performing group after their first two years of life. Fitness may be strongly sex‐specific for both ecological and evolutionary reasons (Clutton‐Brock and Isvaran 2007). In our case, male and female Wandering Albatross may have experienced different ecological conditions due to sex‐specific distribution at sea. For example, females may have been more exposed to fisheries and thus to bycatch mortality than males (Weimerskirch et al. 1997), which could act as a selective force (Barbraud et al. 2013). More importantly, the adult sex ratio of this population is expected to be biased in favor of males (Weimerskirch et al. 2005). In this context, different performance between males and females regarding both reproduction and survival may occur, but could not be associated with individual quality.

Demographic parameter estimates suggested that the level of heterogeneity differed among life‐history traits. In particular, survival heterogeneity was less important compared to reproductive heterogeneity. We suggest that this pattern could be linked to the canalization of life history traits. The canalization theory suggests that natural selection buffers life‐history traits that are most closely related to individual fitness against both genetic and environmental perturbations (Stearns and Kawecki 1994). In long‐lived species, adult survival, which is strongly related to individual fitness, is buffered against temporal variation (Gaillard and Yoccoz 2003). In species with slow life‐history strategies, individuals may skip reproduction when experiencing poor environmental conditions (Cubaynes et al. 2011), or, if breeding is under way, decrease breeding investment, shunting the costs to their offspring to assure their proper maintenance (Navarro and González‐Solís 2007). Thus, the lower heterogeneity observed in survival compared to breeding parameters could result from the canalization of survival to the detriment of breeding investment. This suggests that individuals adopt a prudent strategy regardless their phenotypic characteristic (Jenouvrier et al. 2015) and do not support the existence of different individual reproductive strategies in this species. Furthermore, if this interpretation is correct, it follows that short lived species with faster life‐history strategies should show higher heterogeneity in survival and lower heterogeneity for reproductive process relative to long‐lived species. This prediction, is supported for survival by results from a comparative study on 11 populations belonging to 9 species situated on the slow‐fast continuum (Péron et al. 2016) but needs to be further investigated in future studies.

Although our results strongly support consistent among‐individual differences in demographic rates, a major result of this study is that performance patterns seemed to be reversing in late life. While survival decreased progressively after the age of 30 in both sexes, survival of high quality males, but not high quality females, decreased more strongly compared to low quality individuals. This suggests that the high performance of high quality males, but not females, result in stronger actuarial senescence and confirms the sex‐specific aging pattern previously found in this species (Pardo et al. 2013). Old males, but not old females, make longer trips at sea and fail to restore baseline corticosterone levels, which suggest that their level of stress remains high at old age when foraging (Lecomte et al. 2010). Hence, the performance of high quality individuals could be constrained at older age by a trade‐off between survival and reproduction that was not apparent during most of the adult life. Similarly, high quality female ground squirrels *Tamiasciurus hudsonicus* (Descamps et al. 2006) and high quality male Alpine ibex *Capra ibex* (Toïgo et al. 2013) showing high reproductive rate, suffered lower adult survival than low quality individuals only at the end of life. Individuals having consistently higher demographic performances without apparent short‐term costs may show long term decreasing performances supporting the existence of a trade‐off between early and late life performances (Lemaître et al. 2015). This result also suggests that individual quality could be an important factor to explain among individual variation in senescence rate.

### The origin of individual quality

Studies investigating individual heterogeneity agree that permanent differences among individuals arise from both intrinsic factors (i.e., genetic) and extrinsic factors affecting ontogenetic development (i.e., early‐life environment, parental effects). However, few empirical studies have addressed this issue directly. Here, focusing on the potential effect of early‐life environment, we found that the probability to belong to one of the two quality groups varied over time. Keeping in mind that the two heterogeneity groups used in our finite mixture model are just a modeling approximation to catch unobservable individual quality, which is theoretically a continuous trait, this result suggests that different cohorts, which by definition are born in different years, have individuals of different average quality. This temporal variation lend support to the importance of environmental factors in determining individual quality, as it is the only source of variation expected to generate strong fluctuations over short (yearly) time scales.

Cohort effects lasting until adulthood have been documented in various taxa, including birds (Reid et al. 2003), mammals (Descamps et al. 2008), squamates (Madsen and Shine 2000), and fishes (Baudron et al. 2014). Individuals experiencing favorable early‐life conditions may exhibit higher demographic performance through their life compared to those exposed to poor early‐life conditions (Lindström 1999, Metcalfe and Monaghan 2001). Recently, van Gils et al. (2016) reported morphological variation among cohorts of the Red Knot *Calidris canutus* that could be considered as variation in individual quality. Individuals born during warm years, corresponding to years with low food availability, showed body shrinkage, especially shorter bills, that decreased their foraging abilities on wintering grounds and negatively affected their future survival. Food availability during the ontogenetic period, acting directly or indirectly through parental care, seems to be a key environmental factor determining the quality of a given cohort in birds and mammals (Descamps et al. 2008, Millon et al. 2011), while temperature could also be an important determinant for ectothermic organisms such as fishes (Baudron et al. 2014). These results suggest that beyond direct effects on demography, environmental variations impairing the quality of an entire cohort, may have important delayed effects on population dynamics (Beckerman et al. 2002, Lindström and Kokko 2002).

### Long‐lasting density effect

Results suggest that cohort quality in this Albatross population was related to population density in the year of birth. Individuals born in years of low population density had a higher probability to be assigned to the high quality group, and thus to show high demographic performance throughout their life. This corresponds to a delayed density effect. The *R* ^2^ estimated for this relationship was relatively modest (18%). However, we need to keep in mind that our model, despite its complexity, only provides a rough estimation of individual quality. Furthermore, stochastic processes may determine a part of a life‐history trajectory independently of the intrinsic individual ability to survive and reproduce. This is especially likely for individuals with short lifespans. For this reason, an *R* ^2^ value of 18% could nevertheless indicate that we have identified an important factor.

Relationships between individual quality and population density in the year of birth have already been observed in moths. Wellington (1960) documented that when *Malacosoma pluviale* were overcrowded, emerging larvae were much weaker and sluggish and that this frailty persisted through the larval stage until adult age. Decreasing individual quality seemed an important factor to explain population collapse after an outbreak in the study population. A quality density‐dependent mechanism has also been proposed to drive cyclical population dynamics of six species of Lepidoptera (Ginzburg and Taneyhill 1994). In ungulates, high population density in the year of birth may impair the quality of an entire cohort with visible consequences until adulthood (Bonenfant et al. 2009). Individuals born in cohorts under higher population density were on average lighter at the adult stage, with body mass an important factor for individual fitness in this taxon (Mysterud et al. 2002, Pettorelli et al. 2002). Similarly, Soay sheep *Ovis aries* born under low population density had higher survival probability later in life (Forchhammer et al. 2001). Long‐lived species could be particularly prone to such long‐term density effects since juveniles, i.e., growing individuals, are expected to be the first age class affected by increasing population density (Eberhardt 2002). To our knowledge, this study is the first to suggest long‐lasting population density effects on cohort performance in birds. The underlying process explaining this long‐term effect in this species could be related to parental investment variations depending on intraspecific competition for food during the breeding season. In seabirds, foraging competition among breeders may be important, affecting colony distribution (Furness and Birkhead 1984), foraging trip duration, and efficiency (Lewis et al. 2001, Lescroël et al. 2010). In long‐lived species, life‐history theory predicts that breeders will minimize their energetic costs to preserve future breeding attempts (Stearns 1992). Thus, in cases where increasing breeding population size implies a higher foraging effort, parents may shunt this additional cost on their chick, reducing chick food supply with negative consequences on chick development (Navarro and González‐Solís 2007).

Density effects may induce cohort variations that have double consequences. First, a direct numerical effect (sensu Gaillard et al. 2003) due to decreasing juvenile survival that may induce variation in future recruitment, and second, a long‐term effect through individual quality that affects individual performance throughout life. However, long‐lasting effects are more complex to anticipate than direct numerical effects, since negative consequences at the cohort scale did not occur in every case. By contrast, increasing early‐life mortality under poor environmental conditions may also remove lower quality individuals from the cohort and thus bring together only those individuals with high performance at the adult stage, a process known as viability selection. Based on three ungulate species, Hamel et al. (2009b) concluded that a substantial part of variation in individual quality among cohorts originates from the early‐life environment, but, depending on the species, negative long‐term effect or viability selection was predominantly observed. These two non‐exclusive mechanisms have even been described simultaneously within the same population acting either one or the other preponderantly according to the sex, for example in the roe deer *Capreolus capreolus* (Garratt et al. 2015). In our study, even if viability selection may have taken place owing to heterogeneity in immature survival, results suggest the dominance of negative long‐term effects of the early‐life environment. Indeed, cohorts born under high population density suffered higher juvenile mortality (Fay et al. 2015a) and, contrary to what is expected by viability selection, tended to have lower performances throughout their adult life.

## Conclusion

This study showed that the observed heterogeneity in life‐history trajectories in a Wandering Albatross population could be related to variation in individual quality, i.e., variation in the intrinsic ability to survive and reproduce. Some individuals consistently outperformed others during most of their life, although senescence was stronger in older males with high initial performance. Variation in individual quality seemed strongly affected by extrinsic factors experienced during the ontogenetic period. Results suggested that population density in the year of birth partly explained variation in individual quality leading to population regulation via delayed density effects. This study adds to the emerging view that individual quality, more than a noisy parameter, may be a structuring feature affecting individual life history trajectories and potentially population dynamics.

## References

[ecm1275-bib-0001] Annett, C. A. , and R. Pierotti . 1999 Long‐term reproductive output in Western Gulls: consequences of alternate tactics in diet choice. Ecology 80:288–297.

[ecm1275-bib-0002] Aubry, L. M. , E. Cam , D. N. Koons , J.‐Y. Monnat , and S. Pavard . 2011 Drivers of age‐specific survival in a long‐lived seabird: contributions of observed and hidden sources of heterogeneity: reproduction and survival trade‐offs in the Kittiwake. Journal of Animal Ecology 80:375–383. 21182519 10.1111/j.1365-2656.2010.01784.x

[ecm1275-bib-0003] Authier, M. , L. M. Aubry , and E. Cam . 2017 Wolf in sheep's clothing: model misspecification undermines tests of the neutral theory for life histories. Ecology and Evolution 7:3348–3361. 28515871 10.1002/ece3.2874 PMC5433986

[ecm1275-bib-0004] Barbraud, C. , G. N. Tuck , R. Thomson , K. Delord , and H. Weimerskirch . 2013 Fisheries bycatch as an inadvertent human‐induced evolutionary mechanism. PLoS ONE 8:e60353. 23593199 10.1371/journal.pone.0060353 PMC3622665

[ecm1275-bib-0005] Baudron, A. R. , C. L. Needle , A. D. Rijnsdorp , and C. Tara Marshall . 2014 Warming temperatures and smaller body sizes: synchronous changes in growth of North Sea fishes. Global Change Biology 20:1023–1031. 24375891 10.1111/gcb.12514

[ecm1275-bib-0006] Beckerman, A. , T. G. Benton , E. Ranta , V. Kaitala , and P. Lundberg . 2002 Population dynamic consequences of delayed life‐history effects. Trends in Ecology & Evolution 17:263–269.

[ecm1275-bib-0007] Benton, T. G. , S. J. Plaistow , and T. N. Coulson . 2006 Complex population dynamics and complex causation: devils, details and demography. Proceedings of the Royal Society B 273:1173–1181. 16720388 10.1098/rspb.2006.3495 PMC1560275

[ecm1275-bib-0008] Bergeron, P. , R. Baeta , F. Pelletier , D. Réale , and D. Garant . 2011 Individual quality: Tautology or biological reality?: individual quality. Journal of Animal Ecology 80:361–364. 21054382 10.1111/j.1365-2656.2010.01770.x

[ecm1275-bib-0009] Bergeron, P. , M. Festa‐Bianchet , A. Von Hardenberg , and B. Bassano . 2008 Heterogeneity in male horn growth and longevity in a highly sexually dimorphic ungulate. Oikos 117:77–82.

[ecm1275-bib-0010] Bérubé, C. H. , M. Festa‐Bianchet , and J. T. Jorgenson . 1999 Individual differences, longevity, and reproductive senescence in bighorn ewes. Ecology 80:2555–2565.

[ecm1275-bib-0011] Bolnick, D. I. , P. Amarasekare , M. S. Araújo , R. Bürger , J. M. Levine , M. Novak , V. H. W. Rudolf , S. J. Schreiber , M. C. Urban , and D. A. Vasseur . 2011 Why intraspecific trait variation matters in community ecology. Trends in Ecology & Evolution 26:183–192. 21367482 10.1016/j.tree.2011.01.009 PMC3088364

[ecm1275-bib-0012] Bonenfant, C. , et al. 2009 Empirical evidence of density‐dependence in populations of large herbivores. Advances in Ecological Research 41:313–357.

[ecm1275-bib-0013] Bonnet, T. , and E. Postma . 2016 Successful by chance? The power of mixed models and neutral simulations for the detection of individual fixed heterogeneity in fitness components. American Naturalist 187:60–74. 10.1086/684158 27277403

[ecm1275-bib-0014] Burnham, K. P. , and D. R. Anderson . 2002 Model selection and multimodel inference: a practical information‐theoretic approach. Springer Science & Business Media, New York, USA.

[ecm1275-bib-0015] Cam, E. , L. M. Aubry , and M. Authier . 2016 The conundrum of heterogeneities in life history studies. Trends in Ecology & Evolution 31:872–886. 27665020 10.1016/j.tree.2016.08.002

[ecm1275-bib-0016] Cam, E. , W. A. Link , E. G. Cooch , J.‐Y. Monnat , and E. Danchin . 2002 Individual covariation in life‐history traits: seeing the trees despite the forest. American Naturalist 159:96–105. 10.1086/324126 18707403

[ecm1275-bib-0017] Cam, E. , et al. 2012 Looking for a needle in a haystack: inference about individual fitness components in a heterogeneous population. Oikos 122:739–753.

[ecm1275-bib-0018] Camacho, C. , D. Canal , and J. Potti . 2013 Nonrandom dispersal drives phenotypic divergence within a bird population. Ecology and Evolution 3:4841–4848. 24363908 10.1002/ece3.563 PMC3867915

[ecm1275-bib-0019] Chambert, T. , J. J. Rotella , M. D. Higgs , and R. A. Garrott . 2013 Individual heterogeneity in reproductive rates and cost of reproduction in a long‐lived vertebrate. Ecology and Evolution 3:2047–2060. 23919151 10.1002/ece3.615 PMC3728946

[ecm1275-bib-0020] Charmantier, A. , C. Perrins , R. H. McCleery , and B. C. Sheldon . 2006 Quantitative genetics of age at reproduction in wild swans: support for antagonistic pleiotropy models of senescence. Proceedings of the National Academy of Sciences USA 103:6587–6592. 10.1073/pnas.0511123103 PMC1458927 16618935

[ecm1275-bib-0021] Choquet, R. , J.‐D. Lebreton , O. Gimenez , A.‐M. Reboulet , and R. Pradel . 2009a U‐CARE: utilities for performing goodness of fit tests and manipulating capture–recapture data. Ecography 32:1071–1074.

[ecm1275-bib-0022] Choquet, R. , L. Rouan , and R. Pradel . 2009b Program E‐SURGE: a software application for fitting multievent models Pages 845–865 *in* Thomson D. L., Cooch E. G., and Conroy M. J., editors. Modeling demographic processes in marked populations. Springer, New York, New York, USA.

[ecm1275-bib-0023] Clutton‐Brock, T. H. , and K. Isvaran . 2007 Sex differences in ageing in natural populations of vertebrates. Proceedings of the Royal Society B 274:3097–3104. 17939988 10.1098/rspb.2007.1138 PMC2293943

[ecm1275-bib-0024] Coulson, J. C. 1968 Differences in the quality of birds nesting in the centre and on the edges of a colony. Nature 217:478–479.

[ecm1275-bib-0025] Cubaynes, S. , P. F. Doherty , E. A. Schreiber , and O. Gimenez . 2011 To breed or not to breed: a seabird's response to extreme climatic events. Biology Letters 7:303–306. 20943677 10.1098/rsbl.2010.0778 PMC3061172

[ecm1275-bib-0026] Darwin, C. 1859 The origin of species. J. Murray, London, UK.

[ecm1275-bib-0027] Descamps, S. , S. Boutin , D. Berteaux , and J.‐M. Gaillard . 2006 Best squirrels trade a long life for an early reproduction. Proceedings of the Royal Society B 273:2369–2374. 16928640 10.1098/rspb.2006.3588 PMC1636082

[ecm1275-bib-0028] Descamps, S. , S. Boutin , D. Berteaux , A. G. McAdam , and J.‐M. Gaillard . 2008 Cohort effects in red squirrels: the influence of density, food abundance and temperature on future survival and reproductive success. Journal of Animal Ecology 77:305–314. 18179549 10.1111/j.1365-2656.2007.01340.x

[ecm1275-bib-0029] Desprez, M. , R. Harcourt , M. A. Hindell , S. Cubaynes , O. Gimenez , and C. R. McMahon . 2014 Age‐specific cost of first reproduction in female southern elephant seals. Biology Letters 10:20140264. 24872464 10.1098/rsbl.2014.0264 PMC4046382

[ecm1275-bib-0030] Eberhardt, L. L. 2002 A paradigm for population analysis of long‐lived vertebrates. Ecology 83:2841–2854.

[ecm1275-bib-0031] Fay, R. , C. Barbraud , K. Delord , and H. Weimerskirch . 2016 Variation in the age of first reproduction: Different strategies or individual quality? Ecology 97:1842–1851. 27859167 10.1890/15-1485.1 PMC6681017

[ecm1275-bib-0032] Fay, R. , H. Weimerskirch , K. Delord , and C. Barbraud . 2015a Population density and climate shape early‐life survival and recruitment in a long‐lived pelagic seabird. Journal of Animal Ecology 84:1423–1433. 25976400 10.1111/1365-2656.12390

[ecm1275-bib-0033] Fay, R. , H. Weimerskirch , K. Delord , and C. Barbraud . 2015b Data from: population density and climate shape early‐life survival and recruitment in a long‐lived pelagic seabird. Dryad Digital Repository, 10.5061/dryad.p62h7. 25976400

[ecm1275-bib-0034] Forchhammer, M. C. , T. H. Clutton‐Brock , J. Lindström , and S. D. Albon . 2001 Climate and population density induce long‐term cohort variation in a northern ungulate. Journal of Animal Ecology 70:721–729.

[ecm1275-bib-0035] Furness, R. W. , and T. R. Birkhead . 1984 Seabird colony distributions suggest competition for food supplies during the breeding season. Nature 311:655–656.

[ecm1275-bib-0036] Gaillard, J.‐M. , A. Loison , C. ToÏgo , D. Delorme , and G. Van Laere . 2003 Cohort effects and deer population dynamics. Ecoscience 10:412–420.

[ecm1275-bib-0037] Gaillard, J.‐M. , and N. G. Yoccoz . 2003 Temporal variation in survival of mammals: A case of environmental canalization? Ecology 84:3294–3306.

[ecm1275-bib-0038] Garratt, M. , J.‐F. Lemaître , M. Douhard , C. Bonenfant , G. Capron , C. Warnant , F. Klein , R. C. Brooks , and J.‐M. Gaillard . 2015 High juvenile mortality is associated with sex‐specific adult survival and lifespan in wild roe deer. Current Biology 25:759–763. 25683801 10.1016/j.cub.2014.11.071

[ecm1275-bib-0039] Ginzburg, L. R. , and D. E. Taneyhill . 1994 Population cycles of forest Lepidoptera: a maternal effect hypothesis. Journal of Animal Ecology 63:79–92.

[ecm1275-bib-0040] Griffen, B. D. , and A. P. Norelli . 2015 Spatially variable habitat quality contributes to within‐population variation in reproductive success. Ecology and Evolution 5:1474–1483. 25897386 10.1002/ece3.1427 PMC4395176

[ecm1275-bib-0041] Grosbois, V. , O. Gimenez , J.‐M. Gaillard , R. Pradel , C. Barbraud , J. Clobert , A. P. Møller , and H. Weimerskirch . 2008 Assessing the impact of climate variation on survival in vertebrate populations. Biological Reviews 83:357–399. 18715402 10.1111/j.1469-185X.2008.00047.x

[ecm1275-bib-0042] Hamel, S. , S. D. Côté , J.‐M. Gaillard , and M. Festa‐Bianchet . 2009a Individual variation in reproductive costs of reproduction: high‐quality females always do better. Journal of Animal Ecology 78:143–151. 18700872 10.1111/j.1365-2656.2008.01459.x

[ecm1275-bib-0043] Hamel, S. , J.‐M. Gaillard , M. Festa‐Bianchet , and S. D. Côté . 2009b Individual quality, early‐life conditions, and reproductive success in contrasted populations of large herbivores. Ecology 90:1981–1995. 19694145 10.1890/08-0596.1

[ecm1275-bib-0044] Hamel, S. , N. G. Yoccoz , and J.‐M. Gaillard . 2016 Assessing variation in life‐history tactics within a population using mixture regression models: a practical guide for evolutionary ecologists. Biological Reviews 92:754–775. 26932678 10.1111/brv.12254

[ecm1275-bib-0095] Jenouvrier, S. , L. M. Aubry , C. Barbraud , H. Weimerskirch , and H. Caswell . 2017 Interacting effects of unobserved heterogeneity and individual stochasticity in the life‐history of the Southern fulmar. Journal of Animal Ecology, *in press*. 10.1111/1365-2656.12752 PMC5765524 28886208

[ecm1275-bib-0045] Jenouvrier, S. , C. Peron , and H. Weimerskirch . 2015 Extreme climate events and individual heterogeneity shape life history traits and population dynamics. Ecological Monographs 85:605–624.

[ecm1275-bib-0046] Kendall, B. E. , G. A. Fox , M. Fujiwara , and T. M. Nogeire . 2011 Demographic heterogeneity, cohort selection, and population growth. Ecology 92:1985–1993. 22073789 10.1890/11-0079.1

[ecm1275-bib-0047] Knape, J. , N. Jonzén , M. Sköld , J. Kikkawa , and H. McCallum . 2011 Individual heterogeneity and senescence in Silvereyes on Heron Island. Ecology 92:813–820. 21661544 10.1890/10-0183.1

[ecm1275-bib-0048] Lack, D. 1954 The natural regulation of animal numbers. Clarendon Press, Oxford, UK.

[ecm1275-bib-0049] Lecomte, V. J. , et al. 2010 Patterns of aging in the long‐lived wandering albatross. Proceedings of the National Academy of Sciences USA 107:6370–6375. 10.1073/pnas.0911181107 PMC2852007 20308547

[ecm1275-bib-0050] Lemaître, J.‐F. , V. Berger , C. Bonenfant , M. Douhard , M. Gamelon , F. Plard , and J.‐M. Gaillard . 2015 Early‐late life trade‐offs and the evolution of ageing in the wild. Proceedings of the Royal Society B 282:20150209. 25833848 10.1098/rspb.2015.0209 PMC4426628

[ecm1275-bib-0051] Lescroël, A. , G. Ballard , V. Toniolo , K. J. Barton , P. R. Wilson , P. O. Lyver , and D. G. Ainley . 2010 Working less to gain more: When breeding quality relates to foraging efficiency. Ecology 91:2044–2055. 20715627 10.1890/09-0766.1

[ecm1275-bib-0052] Lewis, S. , T. N. Sherratt , K. C. Hamer , and S. Wanless . 2001 Evidence of intra‐specific competition for food in a pelagic seabird. Nature 412:816–819. 11518965 10.1038/35090566

[ecm1275-bib-0053] Lindberg, M. S. , J. S. Sedinger , and J.‐D. Lebreton . 2013 Individual heterogeneity in black brant survival and recruitment with implications for harvest dynamics. Ecology and Evolution 3:4045–4056. 24324858 10.1002/ece3.767 PMC3853552

[ecm1275-bib-0054] Lindström, J. 1999 Early development and fitness in birds and mammals. Trends in Ecology & Evolution 14:343–348. 10441307 10.1016/s0169-5347(99)01639-0

[ecm1275-bib-0055] Lindström, J. , and H. Kokko . 2002 Cohort effects and population dynamics. Ecology Letters 5:338–344.

[ecm1275-bib-0056] Madsen, T. , and R. Shine . 2000 Silver spoons and snake body sizes: prey availability early in life influences long‐term growth rates of free‐ranging pythons. Journal of Animal Ecology 69:952–958.

[ecm1275-bib-0057] McCleery, R. , C. Perrins , B. Sheldon , and A. Charmantier . 2008 Age‐specific reproduction in a long‐lived species: the combined effects of senescence and individual quality. Proceedings of the Royal Society B 275:963–970. 18230597 10.1098/rspb.2007.1418 PMC2599932

[ecm1275-bib-0058] McNamara, J. M. , and A. I. Houston . 1996 State‐dependent life histories. Nature 380:215–221. 8637568 10.1038/380215a0

[ecm1275-bib-0059] Metcalfe, N. B. , and P. Monaghan . 2001 Compensation for a bad start: Grow now, pay later? Trends in Ecology & Evolution 16:254–260. 11301155 10.1016/s0169-5347(01)02124-3

[ecm1275-bib-0060] Millon, A. , S. J. Petty , B. Little , and X. Lambin . 2011 Natal conditions alter age‐specific reproduction but not survival or senescence in a long‐lived bird of prey: Natal effect and senescence in owls. Journal of Animal Ecology 80:968–975. 21466554 10.1111/j.1365-2656.2011.01842.x

[ecm1275-bib-0061] Moyes, K. , B. J. Morgan , A. Morris , S. J. Morris , T. H. Clutton‐Brock , and T. Coulson . 2009 Exploring individual quality in a wild population of red deer. Journal of Animal Ecology 78:406–413. 19021783 10.1111/j.1365-2656.2008.01497.x

[ecm1275-bib-0062] Mysterud, A. , R. Langvatn , N. G. Yoccoz , and N. C. Stenseth . 2002 Large‐scale habitat variability, delayed density effects and red deer populations in Norway. Journal of Animal Ecology 71:569–580.

[ecm1275-bib-0063] Navarro, J. , and J. González‐Solís . 2007 Experimental increase of flying costs in a pelagic seabird: effects on foraging strategies, nutritional state and chick condition. Oecologia 151:150–160. 17124570 10.1007/s00442-006-0559-0

[ecm1275-bib-0064] Nussey, D. H. , T. Coulson , M. Festa‐Bianchet , and J.‐M. Gaillard . 2008 Measuring senescence in wild animal populations: towards a longitudinal approach. Functional Ecology 22:393–406.

[ecm1275-bib-0065] Oro, D. 2008 Living in a ghetto within a local population: an empirical example of an ideal despotic distribution. Ecology 89:838–846. 18459346 10.1890/06-1936.1

[ecm1275-bib-0066] Pardo, D. , C. Barbraud , and H. Weimerskirch . 2013 Females better face senescence in the wandering albatross. Oecologia 173:1283–1294. 23797411 10.1007/s00442-013-2704-x

[ecm1275-bib-0067] Pardo, D. , C. Barbraud , and H. Weimerskirch . 2014 What shall I do now? State‐dependent variations of life‐history traits with aging in Wandering Albatrosses. Ecology and Evolution 4:474–487. 24634731 10.1002/ece3.882 PMC3936393

[ecm1275-bib-0068] Péron, G. , et al. 2016 Evidence of reduced individual heterogeneity in adult survival of long‐lived species. Evolution 70:2909–2914. 27813056 10.1111/evo.13098

[ecm1275-bib-0069] Pettorelli, N. , J.‐M. Gaillard , G. Van Laere , P. Duncan , P. Kjellander , O. Liberg , D. Delorme , and D. Maillard . 2002 Variations in adult body mass in roe deer: the effects of population density at birth and of habitat quality. Proceedings of the Royal Society B 269:747–753. 11934368 10.1098/rspb.2001.1791 PMC1690952

[ecm1275-bib-0070] Plard, F. , C. Bonenfant , D. Delorme , and J. M. Gaillard . 2012 Modeling reproductive trajectories of roe deer females: Fixed or dynamic heterogeneity? Theoretical Population Biology 82:317–328. 23316493 10.1016/j.tpb.2012.03.006

[ecm1275-bib-0071] Pledger, S. 2005 The performance of mixture models in heterogeneous closed population capture–recapture. Biometrics 61:868–873. 16135042 10.1111/j.1541-020X.2005.00411_1.x

[ecm1275-bib-0072] Pledger, S. , K. H. Pollock , and J. L. Norris . 2003 Open capture‐recapture models with heterogeneity: I. Cormack‐Jolly‐Seber model. Biometrics 59:786–794. 14969456 10.1111/j.0006-341x.2003.00092.x

[ecm1275-bib-0073] Pradel, R. 2005 Multievent: an extension of multistate capture–recapture models to uncertain states. Biometrics 61:442–447. 16011690 10.1111/j.1541-0420.2005.00318.x

[ecm1275-bib-0074] Reid, J. M. , E. M. Bignal , S. Bignal , D. I. McCracken , and P. Monaghan . 2003 Environmental variability, life‐history covariation and cohort effects in the red‐billed chough *Pyrrhocorax pyrrhocorax* . Journal of Animal Ecology 72:36–46.

[ecm1275-bib-0075] Reid, J. M. , E. M. Bignal , S. Bignal , D. I. McCracken , and P. Monaghan . 2006 Spatial variation in demography and population growth rate: the importance of natal location. Journal of Animal Ecology 75:1201–1211. 16922856 10.1111/j.1365-2656.2006.01143.x

[ecm1275-bib-0076] Reznick, D. , L. Nunney , and A. Tessier . 2000 Big houses, big cars, superfleas and the costs of reproduction. Trends in Ecology & Evolution 15:421–425. 10998520 10.1016/s0169-5347(00)01941-8

[ecm1275-bib-0077] Skalski, J. R. 1996 Regression of abundance estimates from mark recapture surveys against environmental covariates. Canadian Journal of Fisheries and Aquatic Sciences 53:196–204.

[ecm1275-bib-0078] Stearns, S. C. 1992 The evolution of life histories. Oxford University Press, Oxford, UK.

[ecm1275-bib-0079] Stearns, S. C. , and T. J. Kawecki . 1994 Fitness sensitivity and the canalization of life‐history traits. Evolution 48:1438–1450. 28568425 10.1111/j.1558-5646.1994.tb02186.x

[ecm1275-bib-0080] Steiner, U. K. , and S. Tuljapurkar . 2012 Neutral theory for life histories and individual variability in fitness components. Proceedings of the National Academy of Sciences USA 109:4684–4689. 10.1073/pnas.1018096109 PMC3311353 22392997

[ecm1275-bib-0081] Toïgo, C. , J.‐M. Gaillard , and A. Loison . 2013 Alpine ibex males grow large horns at no survival cost for most of their lifetime. Oecologia 173:1261–1269. 23774947 10.1007/s00442-013-2700-1

[ecm1275-bib-0082] Tuljapurkar, S. , U. K. Steiner , and S. H. Orzack . 2009 Dynamic heterogeneity in life histories. Ecology Letters 12:93–106. 19016825 10.1111/j.1461-0248.2008.01262.x PMC5662024

[ecm1275-bib-0083] van de Pol, M. , I. Pen , D. Heg , and F. J. Weissing . 2007 Variation in habitat choice and delayed reproduction: Adaptive queuing strategies or individual quality differences? American Naturalist 170:530–541. 10.1086/521237 17891732

[ecm1275-bib-0084] van Gils, J. A. , S. Lisovski , T. Lok , W. Meissner , A. Ożarowska , J. de Fouw , E. Rakhimberdiev , M. Y. Soloviev , T. Piersma , and M. Klaassen . 2016 Body shrinkage due to Arctic warming reduces red knot fitness in tropical wintering range. Science 352:819–821. 27174985 10.1126/science.aad6351

[ecm1275-bib-0085] Van Noordwijk, A. J. , and G. de Jong . 1986 Acquisition and allocation of resources: their influence on variation in life history tactics. American Naturalist 128:137–142.

[ecm1275-bib-0086] Vaupel, J. W. , K. G. Manton , and E. Stallard . 1979 The impact of heterogeneity in individual frailty on the dynamics of mortality. Demography 16:439–454. 510638

[ecm1275-bib-0087] Vaupel, J. W. , and A. I. Yashin . 1985 Heterogeneity's ruses: some surprising effects of selection on population dynamics. American Statistician 39:176–185. 12267300

[ecm1275-bib-0088] Weimerskirch, H. 1992 Reproductive effort in long‐lived birds: age‐specific patterns of condition, reproduction and survival in the wandering albatross. Oikos 64:464–473.

[ecm1275-bib-0089] Weimerskirch, H. , N. Brothers , and P. Jouventin . 1997 Population dynamics of wandering albatross *Diomedea exulans* and Amsterdam albatross *D. amsterdamensis* in the Indian Ocean and their relationships with long‐line fisheries: conservation implications. Biological Conservation 79:257–270.

[ecm1275-bib-0090] Weimerskirch, H. , T. Guionnet , J. Martin , S. A. Shaffer , and D. P. Costa . 2000 Fast and fuel efficient? Optimal use of wind by flying albatrosses. Proceedings of the Royal Society B 267:1869–1874. 11052538 10.1098/rspb.2000.1223 PMC1690761

[ecm1275-bib-0091] Weimerskirch, H. , J. Lallemand , and J. Martin . 2005 Population sex ratio variation in a monogamous long‐lived bird, the wandering albatross. Journal of Animal Ecology 74:285–291.

[ecm1275-bib-0092] Weladji, R. B. , A. Loison , J.‐M. Gaillard , Ø. Holand , A. Mysterud , N. G. Yoccoz , M. Nieminen , and N. C. Stenseth . 2008 Heterogeneity in individual quality overrides costs of reproduction in female reindeer. Oecologia 156:237–247. 18246374 10.1007/s00442-008-0961-x

[ecm1275-bib-0093] Wellington, W. G. 1960 Qualitative changes in natural populations during changes in abundance. Canadian Journal of Zoology 38:289–314.

[ecm1275-bib-0094] Wilson, A. J. , and D. H. Nussey . 2010 What is individual quality? An evolutionary perspective. Trends in Ecology & Evolution 25:207–214. 19897275 10.1016/j.tree.2009.10.002

