# Supplementary material for: From early life to senescence: individual heterogeneity in a long‐lived seabird
Source: Ecol Monogr. 2017 Oct 26;88(1):60–73. doi: 10.1002/ecm.1275 (PMC6084314; doi:10.1002/ecm.1275)
Supplement: Supplementary file 1 [file ECM-88-60-s001.pdf]

Appendix S1

APPROUVÉ

Sex assignment

We used the life histories of 9685 individuals ringed as chicks and resighted between 1965 and 2013. Individuals were sexed in the field based on sexual size and plumage dimorphism, courting and mating behaviours. Since 1999 genetic assignments were available and were systematically used from 2003 (Weimerskirch, Lallemand & Martin 2005). Sex was known for 4913 birds (2431 females and 2482 males), including 2554 from observation (1264 females and 1290 males), and 2359 from genetic (1167 females and 1192 males). Of the remaining unsexed birds, 96.5 % were never seen after fledging on Possession Island, and can be considered as dead before recruitment (Charmantier et al. 2011). As all individuals needed to be included in the model in order to avoid overestimating survival, we inferred the sex of the unsexed birds using a binomial random distribution as in Pardo, Barbraud & Weimerskirch (2013). Knowing that our study population showed an equilibrium sex ratio at fledging (n=3126, p-value=0.99) and that the sex ratio of recaptured birds was also unbiased (n=3085, p-value=0.43), we deduced that the large majority of these birds never seen at the colony were individuals of both sexes in equal proportion. Thus, we were confident that this sex-inference procedure did not introduce spurious patterns since almost all individuals had the same life history (i.e. seen as chick and never recaptured) with a deductible sex-ratio of 1. To valid our process, we repeated the random sex assignment 10 times to check the stability of the sex specific survival parameters.

References

Charmantier, A., Buoro, M., Gimenez, O. & Weimerskirch, H. (2011) Heritability of short-scale natal dispersal in a large-scale foraging bird, the wandering albatross. *Journal of evolutionary biology*, 24, 1487-1496.

Pardo, D., Barbraud, C., & Weimerskirch, H. (2013) Females better face senescence in the wandering albatross. *Oecologia*, 173, 1283-1294.

Weimerskirch, H., Lallemand, J. & Martin, J. (2005) Population sex ratio variation in a monogamous long-lived bird, the wandering albatross. *Journal of Animal Ecology*, 74, 285-291.
