# Supplementary material for: From early life to senescence: individual heterogeneity in a long‐lived seabird
Source: Ecol Monogr. 2017 Oct 26;88(1):60–73. doi: 10.1002/ecm.1275 (PMC6084314; doi:10.1002/ecm.1275)
Supplement: Supplementary file 2 [file ECM-88-60-s002.pdf]

Appendix S2

APPROUVÉ

Bootstrapping method

To estimate breeding probability and breeding success, we needed to combine different estimates of the multi-event model. To obtain 95% confidence intervals of these derived estimates, we used a bootstrapping method based on a multivariate normal distribution. For example, the breeding success is the ratio between the probability to be in success (SB) and the probability to reproduce which is the probability to be in success (SB) plus the probability to be in failure (FB). To estimate the breeding success probability at time t+1 of recruited non breeders (NB) at time t, we used a multivariate normal distribution of two variables corresponding to the average of the two following transitions:

- The probability to be a successful breeder at t+1 ($\psi_{NB}^{NB \to SB}$)

- The probability to be in failure at t+1 ($\psi_{NB}^{NB \to FB}$)

In our model, estimates were not independent. Using the corresponding variance covariance matrix for these two estimates, we sampled a value of each transition and then computed the derived estimate as:

$$\text{Breeding success probability} = \frac{(\psi_{NB}^{NB \to SB})}{(\psi_{NB}^{NB \to SB} + \psi_{NB}^{NB \to FB})}$$

This process is repeated 100000 times and the 95% confidence intervals of the derived parameter were estimated with the quantiles 0.05 and 0.95 of the resulting distribution.
