# Supplementary material for: From early life to senescence: individual heterogeneity in a long‐lived seabird
Source: Ecol Monogr. 2017 Oct 26;88(1):60–73. doi: 10.1002/ecm.1275 (PMC6084314; doi:10.1002/ecm.1275)

APPROUVÉ

Appendix S3

Figure S1. Life cycle graph representing transitions between observable (white) or unobservable states (grey). All birds are ringed as chicks, thus individuals start in the Pre-Recruitment state (PrR). After fledging, all birds remain at least two years continuously at sea (i.e. juvenile stage). Pre-Recruitment state becomes observable from 3 years-old when birds start to return at the colony and are now considered as immature. From 6 years-old, birds can pass into the breeding group of the population though recruitment represented by dashed arrows. Then mature birds irrespectively of age can transit between mature states: successful breeder (SB), failed breeder (FB), recruited non breeder (NB), post successful breeder (PSB) and post failed breeder (PFB).

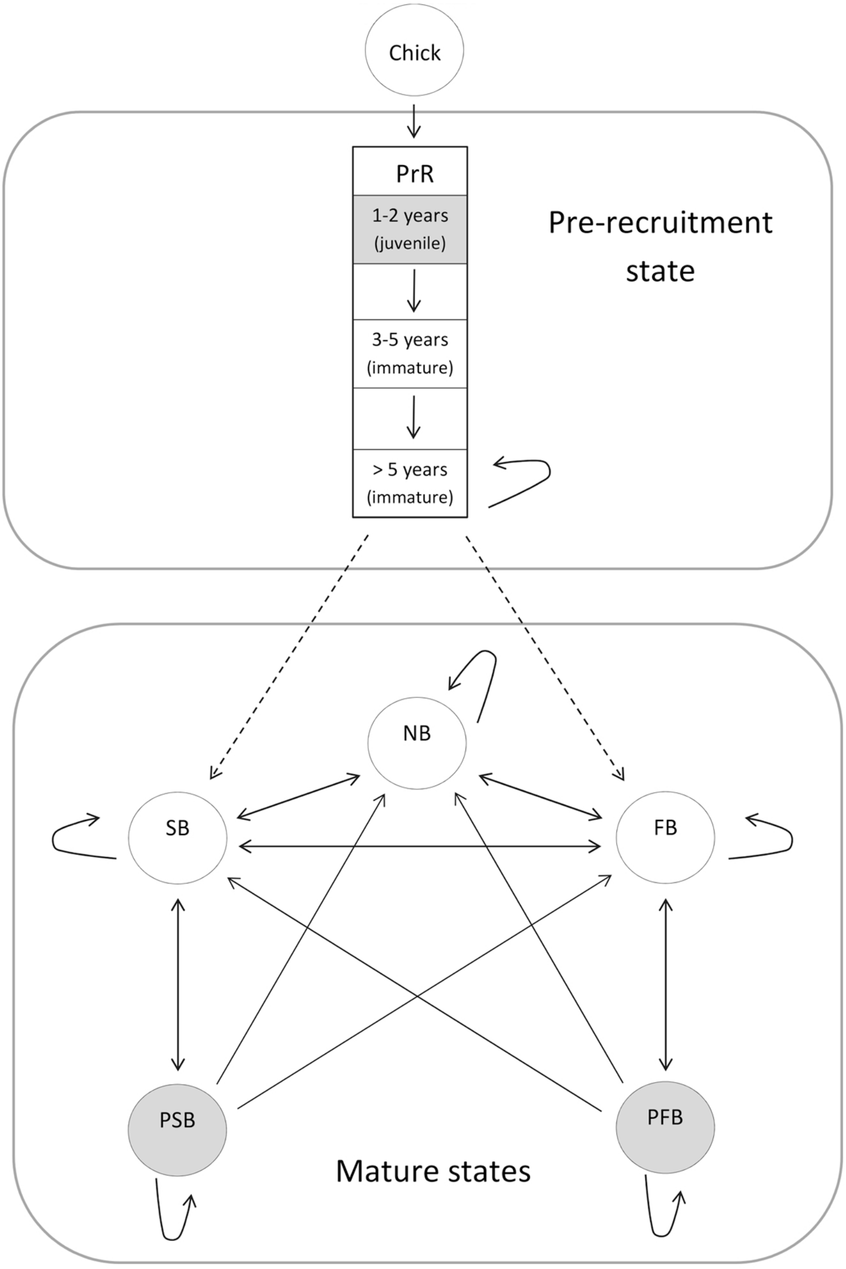

Figure S2. Heterogeneity of age-specific probability of recruitment for (a) females and (b) males of the wandering albatross population of Crozet. Estimates (±SE) came from model 4 (Table 1).

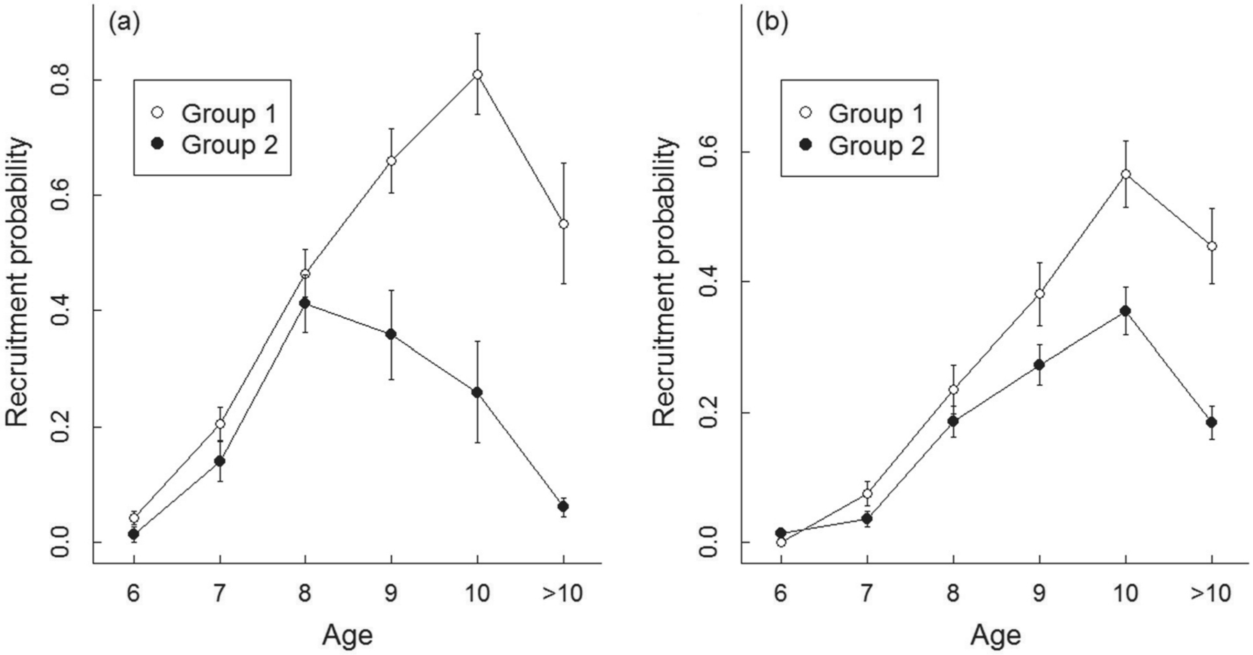

Figure S3. Relationship between the cohort-specific probabilities to belong to group 2 and population density the year of birth for the wandering albatrosses population of Crozet. Estimates of cohort-specific transition (open circles ±SE) were obtained from the additive cohort dependent model (Table 1, M4). Cohort-specific transition probability in group 2 was modelled as a function of population density (dotted line, Table 4, Model 1, P_{Anodev}=0.04).

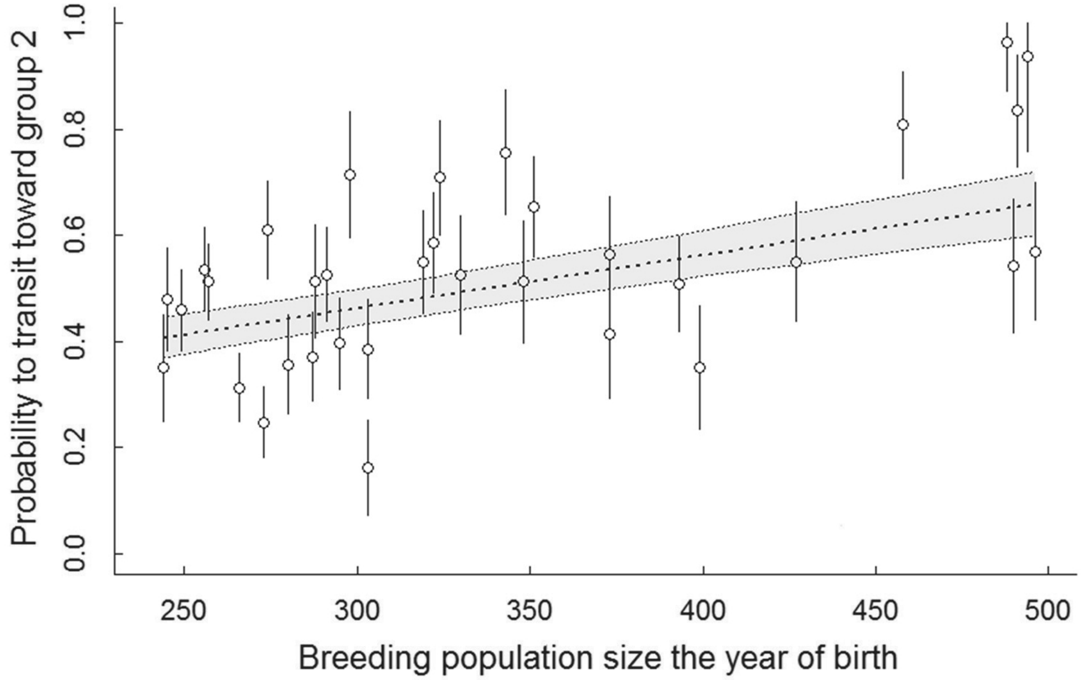

Supplement: Supplementary file 3 [file ECM-88-60-s003.pdf]
